# Modified tectonic keratoplasty with minimal corneal graft for corneal perforation in severe Stevens - Johnson syndrome: a case series study

**DOI:** 10.1186/1471-2415-14-97

**Published:** 2014-08-08

**Authors:** Fuhua Wang, Suxia Li, Ting Wang, Hua Gao, Weiyun Shi

**Affiliations:** 1 Shandong Eye Hospital, Shandong Eye Institute, Shandong Academy of Medical Sciences, 372 Jingsi Road, Jinan 250021, P.R. China

**Keywords:** Corneal transplantation, Corneal perforation, Stevens-Johnson syndrome, Conjunctival flap

## Abstract

**Background:**

Corneal perforation in severe Stevens-Johnson syndrome (SJS) presenting great therapeutic difficulties, the imperative corneal transplantation always result in graft failure and repeated recurrence of perforation. The aim of this study was to evaluate the effectiveness of a modified small tectonic keratoplasty (MSTK) with minimal corneal graft in the management of refractory corneal perforation in severe SJS.

**Methods:**

Refractory corneal perforations in ten patients (10 eyes) with severe SJS were mended with a minimal corneal patch graft, under the guidance of anterior chamber optical coherence tomography, combined with conjunctival flap covering. The outcome measures included healing of the corneal perforation, survival of the corneal graft and conjunctival flap, relevant complications, and improvement in visual acuity.

**Results:**

Corneal perforation healed, and global integrity was achieved in all eyes. No immune rejection or graft melting was detected. Retraction of conjunctival flap occurred in one eye, which was treated with additional procedure. Visual acuity improved in six eyes (60%), unchanged in three eyes (30%) and declined in one eye (10%).

**Conclusions:**

The MSTK combined with conjunctival flap covering seems to be effective for refractory corneal perforation in severe SJS.

## Background

Severe Stevens-Johnson syndrome (SJS) usually affects the eyes with extensive area of necrosis of the ocular surface, which could result in corneal ulcer or even perforation [1-3]. Because of severe dry eye, limbal stem cell deficiency, persistent inflammation, trichiasis and symblepharon that result from the ocular surface cicatrization [4,5], corneal perforation in these patients is usually refractory. Amniotic membrane transplantation (AMT), conjunctival flaps, and bandage contact lens seems does not work well [6-8]. In most cases, corneal transplantation has to be resort to for tectonic reasons.

Orthotopic corneal transplantation is commonly used in treating corneal perforation. But in eyes with severe ocular surface disorders, persistent epithelial defects can lead to graft melting, repeated recurrence of perforation or even loss of vision [9]. Even though temporary success can be achieved in a few cases, long-term immunosuppressive drug administration has to be dependent on, and the graft still faces the risk of failure and other post-operative complications following orthotopic penetrating keratoplasty [1,10]. Hence, preservation of the globe is the main objective of corneal transplantation for severe SJS, and visual rehabilitation becomes an important but secondary objective.

Small corneal graft has been successfully used in treating peripheral corneal perforations as it faces less risks of graft failure [11-14]. Portnoy et al. mentioned in a review that an appropriate size corneal graft could be used to patch the corneal lesions, and then the graft was covered with a conjunctival flap. But no details of the technique was described, and the long-term results were uncertain [15]. In severe dry eye, small grafts still face the challenge of melting and recurrence of corneal perforation [12]. To further facilitate survival of small corneal graft so that it could be successfully applied to the management of corneal perforation in eyes with severe SJS, we contrived to minimize the size of corneal graft and a conjunctival flap was used in a combined procedure. We report here our first experience in a small series of cases.

## Methods

### Patients

The study was approved by the Institutional Review Board of Shandong Eye Hospital and was conducted in accordance with the principles of the Declaration of Helsinki. Possible benefits and risks were explained to all patients, and informed consent was obtained.

The medical records of ten patients (10 eyes), with refractory corneal perforations resulting from severe SJS, who were treated with modified small tectonic keratoplasty (MSTK) combined with conjunctival flap covering were retrospectively reviewed. All the perforations were conducted at Shandong Eye Hospital of Shandong Eye Institute between July 2008 and June 2011. The diagnosis of SJS was mainly based on a history of severe inflammation of the oral, dermal, and tracheal mucous membranes after taking medicine or having an infection [16]. Corneal perforations were confirmed by fluorescent stream tests. The size of corneal perforations and the surrounding corneal lesions was measured by slit-lamp microscopy or high-definition optical coherence tomography (HD-OCT, Carl Zeiss AG, Dublin, USA). All eyes underwent corneal scraping with care under a surgical microscope and topical anesthesia. The biopsy specimens were gram stained and examined under a microscope for possible bacterial and fungal infections. As aqueous leakage could disturb the results of the Schirmer test, it was performed on the fellow eye in all patients. Localization of corneal perforation, synechiae, flat anterior chamber, and visual acuity before surgery were also recorded.

All the patients were immediately administered with ofloxacin eyedrops (Santen, Osaka, Japan) twice per hour at the first presentation. Combined MSTK and conjunctival flap covering was performed within 24 hours. The resected tissues were subjected for etiological examination.

### Surgical procedure

#### Corneal trephination

**Figure 1:**
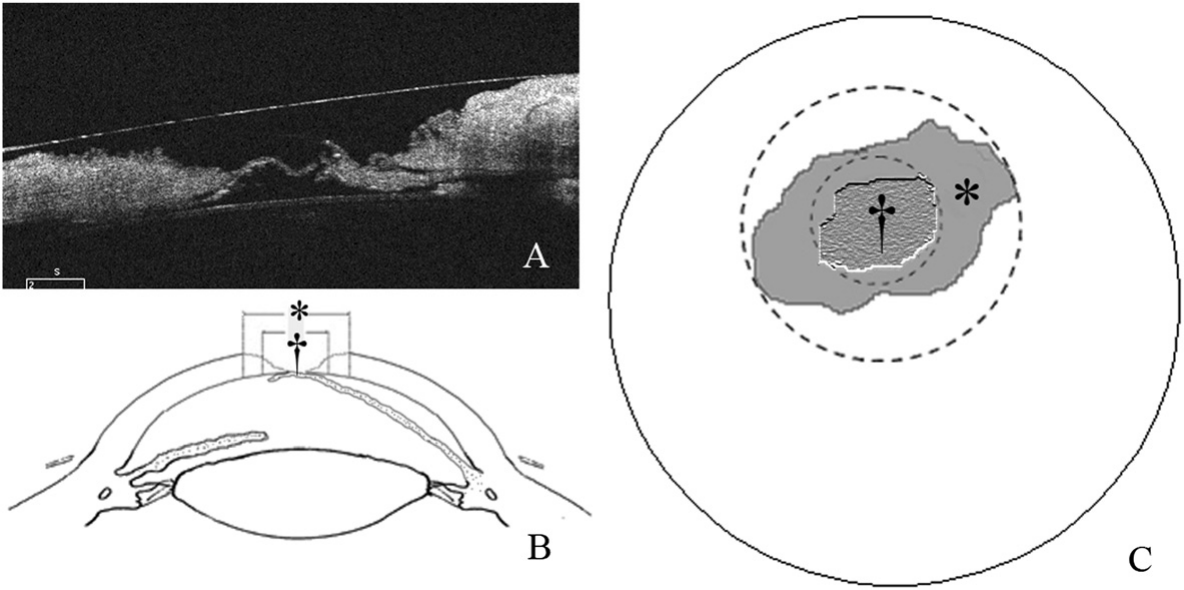
**Determination of trephine diameter. (A)** The appearance of the corneal ulcer and perforation on HD-OCT examination. **(B and C)** Shape of the corneal ulcer (*) and the area with the remaining stroma more than two-thirds of the cornea in thickness (†) in the corneal section and overall appearance. The diameter of the trephine should equal to that of “†”.

The trephine size was determined as 1.0 mm larger than that of the corneal perforation according to the surgeon’s experience for the first two patients. From the third patient, HD-OCT was used to minimize the size of corneal trephine just as illustrated in Figure 1. An area of the corneal lesions, in which the remaining stroma was at least 2/3 of the corneal thickness, was firstly marked out. The trephine diameter was then determined corresponding to the shortest path across the marked area.

#### Recipients

**Figure 2:**
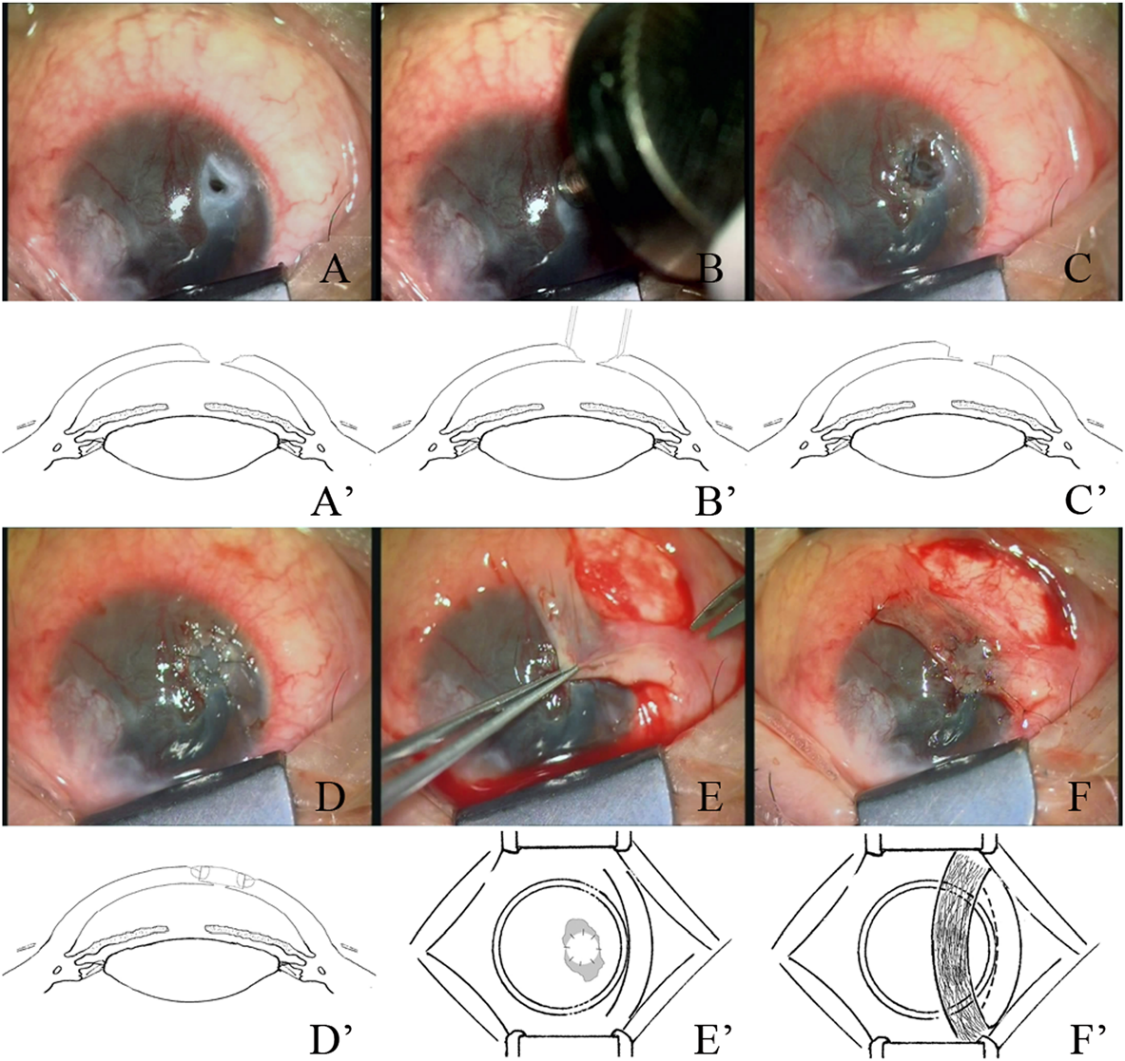
**The procedure of modified small tectonic keratoplasty and conjunctival flap covering. (A and A’)** Corneal ulcer after clearing of necrotic tissues and restoring of synechia. **(B and B’)** A selected trephine was used to mark around the perforation. **(C and C’)** The recipient bed with the posterior stroma and Descemet’s membrane reserved. **(D and D’)** The corneal button was placed in the bed and sutured with interrupted 10–0 nylon sutures. **(E and E’)** A bridge-like conjunctival flap, 0.5 mm larger than the ulcer, was obtained. **(F and F’)** conjunctival flap was secured tightly using 10–0 monofilament nylon sutures.

All surgery was performed under retrobulbar anesthesia by the same surgeon. Necrotic tissues were cleared from the surface of the corneal ulcer. Viscoelastic materials were then injected from the perforation (or, in cases with synechiae, from a corneal paracentesis at the opposite side of the perforation) to maintain the anterior chamber at a safe depth. Meanwhile, the iris incarcerated in the perforation was restored (Figure 2A and A’). The corneal trephine was used to mark around the perforation, and the tissues within the mark were then removed using a diamond blade (Figure 2B and B’). The posterior stroma and Descemet’s membrane were reserved under the precondition that necrotic tissues were completely removed (Figure 2C and C’).

#### Corneal grafts

A full-thickness corneal button was obtained from a cryopreserved donor cornea using a corneal trephine with the same diameter as (when the recipient bed was ≤3 mm) or 0.25 mm larger than (when the recipient bed was >3 mm) the recipient bed (Table 1). The donor button was fixed to recipient with interrupted 10–0 monofilament nylon sutures (Figure 2D and D’).

**Table 1:** Patient demographics, pre- and postoperative data, and follow-up

**Patient number**	**1**	**2**	**3**	**4**	**5**	**6**	**7**	**8**	**9**	**10**
Sex/age (years)	F/48	M/65	M/42	F/54	M/47	M/61	M/54	F/37	F/58	M/22
Schirmer test (mm)	0	1	1	0	1	3	1	2	0	0
Localization of ulcer	PC	CT	PP	PC	PC	PP	PC	PP	PC	PP
Co-morbidities	Trichiasis, symblepharon	Trichiasis, symblepharon	Symblepharon, synechia	Trichiasis, symblepharon	Trichiasis	Trichiasis, symblepharon	Trichiasis, synechia	Synechia	Trichiasis, symblepharon	Synechia
Activity of SJS	Inactive	Active	Active	Active	Active	Inactive	Inactive	Active	Inactive	Active
Prior corneal surgical procedures (times)	AMT(2), CP (1)	CP (2)	AMT(1), CP (1)	CP (1)	CP(1), AMT(1)	AMT(1), LK (3)	LK (2), PK (1)	CP (2)	AMT(1), CP(1)	AMT(1)
Size of corneal lesion (mm)	4.0 × 5.5	6.0 × 7.5	4.5 × 7.0	4.5 × 5.75	5.0 × 6.0	3.5 × 5.0	3.0 × 5.5	3.5 × 5.75	4.0 × 6.5	3.25 × 4.0
Size of perforation (mm)	2.5	3.5	3.0	2.5	2.5	2.0	2.5	2.5	3.0	2.0
Size of corneal graft (mm)	3.75	4.75	4.25	3.75	4.0	3. 5	3.0	3.5	4.0	3.0
Follow-up (months)	38	34	32	32	20	15	14	11	9	6
Preoperative BCVA	20/200	HM/10 cm	CF/10 cm	20/60	HM/50 cm	CF/10 cm	HM/30 cm	20/100	CF/20 cm	20/200
Final BCVA	20/200	20/50	20/100	20/40	HM/30 cm	CF/30 cm	HM/30 cm	20/100	20/200	20/100
Preoperative IOP (mmHg)	5	3	8	3	5	4	11	7	4	8
Final IOP (mmHg)	12	13	16	10	12	19	17	11	15	13

F = Female; M = Male; AMT = Amniotic Membrane Transplantation; CP = Conjunctival Flap; LK = lamellar Keratoplasty; PK = Penetrating Keratoplasty; PC = Paracentral; CT = Central; PP = Peripheral; BCVA = best-corrected visual acuity; HM = Hand Motions; CF = Counting Fingers; IOP = Intraocular Pressure.

#### Conjunctival flaps

The bulbar conjunctiva near corneal lesions with good elasticity and rich blood vessels was selected as the donor site of conjunctival flap. After subconjunctival injection of 0.5 to 1 mL of 2% lidocaine hydrochloride, a double pedicle conjunctival flap, 1 mm wider than the corneal lesion, was obtained 2 mm away from the limbus (Figure 2E and E’). A thin fascia under the conjunctival flap was preserved to avoid injury to conjunctival vessels during separation. Meanwhile, the preserved fascia may also promote tight adhesion of the conjunctival flap to corneal lesions. The conjunctival flap was fully separated toward both ends until it could be pulled to the corneal lesions without tension, and the eyeball could move freely towards all directions. Finally, the conjunctival flap was rotated to cover the entire corneal lesions. A 10–0 monofilament nylon suture was used to secure the flap with a certain tension (Figure 2F and F’). Any effusion beneath the conjunctival flap should be drive out with a muscle hook so that the flap could closely attach to corneal lesions.

### Postoperative treatment and follow-up

Postoperatively, all patients were given intravenous hydrocortisone (2 mg/kg) daily for 3 days. TobraDex eyedrops (Alcon, Fort Worth, TX) and 1% cyclosporine A (North China Pharmaceutical Group, Shijiazhuang, China) were administered four times daily for the first month and tapered thereafter. Preservative-free artificial tears were administered 6 ~ 8 times daily according to the ocular surface conditions.

The patients were followed up weekly for 4 weeks and monthly or bimonthly thereafter. Visual acuity, slit lamp appearance, and intraocular pressure (IOP) were evaluated. All complications were recorded and disposed. Surgical success was defined as healing of the ulcer and perforation, and survival of the conjunctival and corneal grafts. Failure was defined as corneal graft melting, recurrence of an ulcer or perforation in the original position.

### Statistical analysis

SPSS 16.0 was used for statistical analysis. The change in BCVA was analyzed using the Wilcoxon signed-rank test. P values of less than 0.05 were considered statistically significant.

## Results

**Figure 3:**
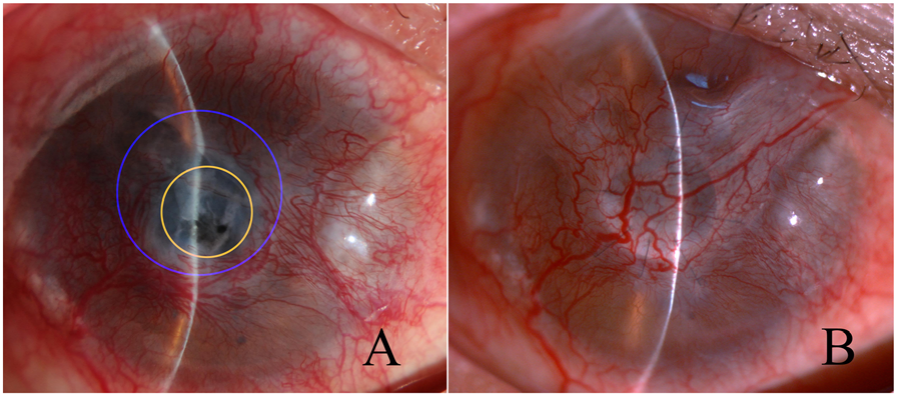
**Preoperative and postoperative corneal perforation in patient 2. (A)** Preoperatively, the size of the maximum diameter(blue) of the corneal ulcer was much larger than that of the minimum diameter(yellow) of the area that a corneal transplantation could be easily performed. **(B)** The corneal perforation healed and the conjunctival flap presented significant regression 6 months after surgery.

The patients were 4 females (40%) and 6 males (60%), with an average age of 48.8 years (range, 22–65 years). The maximum size of each corneal perforation ranged from 2.0 mm to 4.0 mm. There was an irregular corneal ulceration with the outer diameter of 4.0 mm to 7.5 mm around all the perforations (Figure 3A). All the patients had been treated with multiple drugs consisting mainly of local antibiotics and artificial tears, and systemic antibiotics before referred to our institution. Also, conjunctival flap, AMT, penetrating keratoplasty (PK) or/and lamellar keratoplasty had been performed in other hospitals. The clinical characteristics of the patients are summarized in Table 1.

The mean follow-up period was 21.1 ± 11.8 (SD) months (range, 6 to 38 months). Corneal perforations were healed, and the integrity of the globes was restored in all patients (Table 1). No pathogens were detected in any of the corneal scrapings for bacterial and fungal culture.

### Conjunctival flaps

The conjunctival flap showed good adhesion to corneal lesions in 9 patients (patients 1–5, 7–10) during the follow-up period. A progressive regression of conjunctival flap was observed at 1 month after surgery, and the conjunctival flap became semi-transparent when healing of the corneal lesions was completed (Figure 3B). In one patient (patient 6), the conjunctival flap slipped off the corneal lesion and was difficult to be reset to the corneal surface because of poor flexibility associated with an anamnesis of ocular alkali burn. To promote corneal epithelial repair and prevent corneal graft autolysis, conjunctival flap resuture combined with tarsorrhaphy was performed. The corneal lesions were then healed completely.

### Corneal grafts

**Figure 4:**
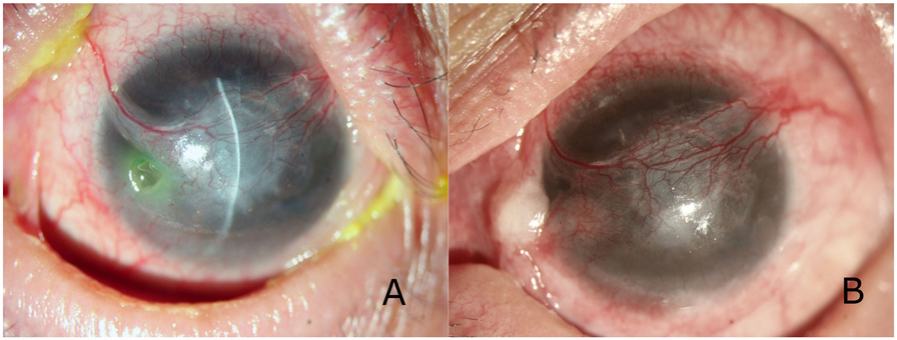
**Postoperative corneal perforation in patient 2. (A)** The corneal perforation healed yielding a visual acuity of 20/200 at 6 months after surgery. But a clean, paranasal corneal ulcer, 2 mm in diameter, occurred just beside the conjunctival flap. **(B)** Both the corneal ulcer and the perforation healed with good survival of the corneal graft and conjunctival flap at 32 months after the second surgery.

The corneal grafts presented mild edema within the first month and became semi-transparent ever since (Figure 3B). No immune rejection and autolysis occurred in any of the patients. A corneal ulcer, 2 mm in diameter, occurred just beside the conjunctival flap in patient 1 at 6 months (Figure 4A). Another conjunctival flap procedure was performed after 1 week of ineffective drug therapy. The perforations healed, and the corneal graft remained stable in the next 32 months follow-up (Figure 4B).

### Visual outcomes and IOP

Visual acuity was improved in 6 eyes (60%), unchanged in 3 eyes (30%), and declined in one eye (10%) at the final follow-up. There was a significant difference between the preoperative and postoperative visual acuities (P = 0.043). The IOP of all the patients was within the normal range throughout the study period.

## Discussion

Because of prolonged ocular surface morbidities, corneal perforations in severe SJS still face great challenge of recurrence, and the final visual function is always pessimistic. In this series of 10 patients, the corneal perforations were successfully repaired with MSTK and conjunctival flap in 9 patients. Tarsorrhaphy was required for remedial reason in one patient with conjunctival flap failure to adhere to the corneal lesion. All corneal grafts remained stable during the follow-up of no less than 1 year, and improved vision was acquired in more than 50% of the cases.

Efficacy of AMT in treating small corneal perforation has been well established but unable to promote corneal stability in patients with severe corneal thinning [17]. Also, because the amniotic membrane cannot control corneal melting effectively in dry eyes [18], it does not seem to work well in patients with SJS [6,10], which was further confirmed in 6 patients in this study. Conjunctival flaps could be used for small eccentric corneal perforations [1], but could not seal large corneal perforations associated with corneal melting because the leap would continue under the flap [19,20]. Things were the same for 7 patients in this study. Tissue adhesives are effective for corneal perforations in a diameter of less than 2 mm but not for those chronic, deep ulcers, and the toxicity may worsen inflammatory reaction [21].

Traditional orthotopic PK, using a large-diameter graft, could achieve comparable anatomical and functional success in treating corneal perforation. But in patients with severe SJS, the corneal grafts are facing high risk of immune rejection, persistent epithelial defection, infection, graft melting, and resultant recurrence of corneal perforation [22]. Tugal-Tutkun et al. reported PK, with grafts of 7.5 mm to 14 mm, for cicatrizing conjunctival diseases in 13 eyes, among which three out of four grafts in patients with SJS failed and required additional procedures [9]. Theoretically, the smaller the size of a corneal graft, the greater is the chance of its survival. Soong et al. reported 3 cases of fistulous wound leaks after cataract surgery successfully treated with small diameter corneal grafts for tectonic reasons [11]. Similar procedures were performed by Chern in treating various peripheral corneal disorders with small-diameter, round, eccentric PK [12]. We previously modified this technique by preserving the deep stroma, Descemet’s membrane, and the endothelium of the corneal bed as much as possible, so that glycerin-cryopreserved grafts could be used with ultimate transparency [13]. But all the corneal grafts used in the reported cases had the same size as corneal lesions, and all the eyes had a relative fertile ocular surface. The single case with ocular surface disorder reported by Chern et al. developed epithelial keratopathy and required further treatment [12]. In this study, two patients had been treated with repeated small corneal graft transplantation, but both failed in graft melting and recurrence of corneal lesions. Therefore, small corneal grafts in patients with severe ocular disorders still risk graft failure.Corneal perforations in SJS are usually developed from corneal melting, and there is commonly an irregular corneal lesion, much larger than corneal perforation, which is usually apparent on HD-OCT examination (Figure 1A). If corneal grafts are prepared according to the corneal ulcer, certainly much larger than the perforation, the risk of graft failure would inevitably increase. In view of the characteristics of corneal perforation in SJS, we hypothesized that the diameter of corneal graft could be further reduced just to repair the perforation. In our experience, grafts can be well sutured as long as the thickness of the edge of the recipient bed is more than two-thirds of the cornea. Hence, instead of trying to cover the entire ulcer, we selected a trephine according to the shortest path through the central ulcer in which the remaining stromal thickness was more than two-thirds of the cornea (Figure 1B and C). In this study, all the 10 corneal grafts were successfully transplanted to the recipients, which indicate the feasibility of corneal transplantation with a reduced-size corneal graft.

Due to the fact that the corneal graft was much smaller than the ulcer, a ‘ditch’ was left around the graft (Figure 1C*). The epithelium of the recipient was expected to step across the ditch to cover the graft, which was a difficult task in the eye with severe ocular surface disorder. To prevent secondary infection and to enable repair of the ditch between the graft and the corneal bed, an effective epithelial barrier must be provided. Also, a sufficient source of nutrients was necessary to ensure the survival of corneal graft. AMT, with the function of promoting epithelialization and decreasing inflammation, neovascularization and fibrosis [17], was a potential selection. But its function would disappear with the amniotic membrane melting within one month after transplantation. Tarsorrhaphy could improve re-epithelialization and prevent corneal melting [23], but it is not acceptable for most patients for poor cosmetic appearance. Moreover, it could interfere with the observation of corneal lesions. So tarsorrhaphy was selected only when there were no other choices. Conjunctival flap, rich in blood vessels, is effective in treating refractory corneal ulcers. Although cosmetic appearance was poor over a period of time after surgery, the conjunctival flap would have a marked regression after healing of corneal lesions [24], and there is not any difference between conjunctival flap blood vessels and corneal neovascular results from severe SJS. Ultimately, the cosmetic appearance and impairment of corneal clarity after conjunctival flap covering are acceptable for most patients. Therefore, in this study, conjunctival flap was used for protective reasons. No one complained about the cosmetic appearance of conjunctival flap.

In this case series, MSTK combined with conjunctival flap achieved favorable effects. The success of this combined procedure may be attributed to the following aspects. First, the corneal graft was quite small, which resulted in a lower risk of immune rejection and melting. Second, the corneal graft restored the integrity of the eye and added a rigid barrier between the aqueous humor and the conjunctival flap, which facilitated adherence of conjunctival flap to the corneal lesions. Third, the conjunctival flap helped to prevent secondary infection by covering the space around the small-diameter corneal graft. Fourth, the flap aided in the absorption of necrotic tissues and promoted vascularization of the wounded cornea [24], thus preventing graft melting and the extension of remaining ulcers. In the only case with conjunctival flap failure, corneal epithelial reconstruction was delayed, and an additional procedure was required, which indicates the importance of conjunctival flap in this combined procedure.

Due to the complexity of corneal perforations in SJS, most patients may have poor visual acuity after treatment, and additional treatment for improving eyesight can be performed. Moreover, conjunctival flaps could not be obtained for protective necessities in patients who do not have enough healthy conjunctivas. Hence, permanent tarsorrhaphy would eventually be resorted to.

## Conclusion

In summary, combined MSTK and conjunctival flap covering is effective in treating corneal perforations in SJS. The eye globe could achieve tectonic integrity with a minimal corneal allograft under the protection of conjunctival flap, and the vision could be partly restored. This combined procedure might also be considered for the treatment of refractory corneal perforations associated with other severe systemic immune diseases.

## Competing interests

The authors declare that they have no competing interests.

## Authors’ contributions

WS designed the study, performed all the operations, participated in interpretation of the data and revised the manuscript critically for important intellectual content. FW, SL and HG assisted in surgeries and overall management of the patients, participated in the data acquisition and analysis. FW also drafted the manuscript and revised it. TW participated in the data acquisition and analysis, contributed to manuscript revision. All authors read and approved the final manuscript.

## Pre-publication history

The pre-publication history for this paper can be accessed here:

http://www.biomedcentral.com/1471-2415/14/97/prepub
